# Unique Functional Abnormalities in Youth with Combined Marijuana Use and Depression: An fMRI Study

**DOI:** 10.3389/fpsyt.2014.00130

**Published:** 2014-09-26

**Authors:** Kristen A. Ford, Michael Wammes, Richard W. Neufeld, Derek Mitchell, Jean Théberge, Peter Williamson, Elizabeth A. Osuch

**Affiliations:** ^1^Department of Psychiatry, University of Western Ontario, London, ON, Canada; ^2^Department of Psychology, University of Western Ontario, London, ON, Canada; ^3^Department of Program in Neuroscience, University of Western Ontario, London, ON, Canada; ^4^Department of Medical Biophysics, University of Western Ontario, London, ON, Canada; ^5^Department of Medical Imaging, University of Western Ontario, London, ON, Canada; ^6^Lawson Health Research Institute, London, ON, Canada; ^7^Department of Diagnostic Imaging, St. Joseph’s Hospital, London, ON, Canada

**Keywords:** reward, cannabis, major depressive disorder, adolescent, young adult, mental health

## Abstract

Prior research has shown a relationship between early onset marijuana (MJ) use and depression; however, this relationship is complex and poorly understood. Here, we utilized passive music listening and fMRI to examine functional brain activation to a rewarding stimulus in 75 participants [healthy controls (HC), patients with major depressive disorder (MDD), frequent MJ users, and the combination of MDD and MJ (MDD + MJ)]. For each participant, a preferred and neutral piece of instrumental music was determined (utilizing ratings on a standardized scale), and each completed two 6-min fMRI scans of a passive music listening task. Data underwent pre-processing and 61 participants were carried forward for analysis (17 HC, 15 MDD, 15 MJ, 14 MDD + MJ). Two statistical analyses were performed using SPM8, an analysis of covariance with two factors (group × music type) and a whole brain, multiple regression analysis incorporating two predictors of interest [MJ use in past 28 days; and Beck Depression Inventory (BDI) score]. We identified a significant group × music type interaction. *Post hoc* comparisons showed that the preferred music had significantly greater activation in the MDD + MJ group in areas including the right middle and inferior frontal gyri extending into the claustrum and putamen and the anterior cingulate. No significant differences were identified in MDD, MJ, or HC groups. Multiple regression analysis showed that activation in medial frontal cortex was positively correlated with amount of MJ use, and activation in areas including the insula was negatively correlated with BDI score. Results showed modulation in brain activation during passive music listening specific to MDD, frequent MJ users. This supports the suggestion that frequent MJ use, when combined with MDD, is associated with changes in neurocircuitry involved in reward processing in ways that are absent with either frequent MJ use or MDD alone. This could help inform clinical recommendations for youth with MDD.

## Introduction

Survey, behavioral, translational, and brain imaging research has found, to varying degrees, a relationship between marijuana (MJ) use and mental illness. However, this relationship is complex and poorly understood. Research shows that 20% of people with a mental health issue have a co-occurring substance use problem, often involving cannabis (1). Recreational MJ use is increasing in prevalence in adolescence and now is even more pervasive than cigarette smoking in youth (2). Additionally, a survey of 12th grade students showed that the lifetime prevalence of MJ use was 36% (3). Studies have shown that cannabis use can precipitate the onset of mood disorders (and psychosis) in vulnerable youth (4), and a growing body of research suggests an association between cannabis use and the onset of mood disorders, especially in youth (5–8). Thus, examining the relationship between MJ use and mental illnesses such as mood disorders is vital in order to mitigate risk to youth. This is especially important since the onset of most mood disorders is before the age of 25 (9), which is when youth are most likely to experiment with marijuana (2).

Due to the prevalence of common depressive symptoms in people who engage in frequent MJ use, such as anhedonia and lack of motivation (10, 11), the neurocircuitry of reward processing may be affected in both populations (10, 12, 13). A number of studies have found abnormalities in the ventral striatum in both adults and youth suffering from depression (14–18). Acute MJ use is associated with increased regional cerebral blood flow in areas known to be involved in reward processing such as ventral and medial frontal cortices, insula, anterior cingulate cortices, and a variety of subcortical regions (19–21). A meta-analysis of 43 studies of MJ use showed a wide range of alterations in brain activation suggesting a compensatory response to chronic cannabis exposure and abnormalities in prefrontal cortex (PFC), orbital frontal cortex, ventral striatum, thalamus, pons, and other reward-processing regions (22). Chronic cannabis use has been shown to affect the brain’s capacity for dopamine synthesis, an alteration related to schizophrenia and psychosis (23) and widely implicated in depressive symptomatology (24, 25). Dopamine is one of the key neurotransmitters involved in reward processing (26), and alterations in this neurotransmitter system during brain development, including the adolescent years, may have longitudinal implications for the rest of the individual’s life.

A variety of stimuli have been utilized in the investigation of the neural circuitry underlying reward processing. Passive music listening paradigms have been shown to strongly modulate activity in reward-related brain regions (27, 28) without involving a cognitive processing component such as decision making. Both MJ use and MDD have been shown to affect cognitive functioning in youth (29, 30), and these deficits may lead to difficulty interpreting paradigms involving both cognitive processing and reward processing. In this study, we utilized a passive music listening paradigm as a stimulus for reward processing without demand on cognitive functioning, and fMRI to examine the neurocircuitry of reward processing in late adolescents/young adult groups of healthy controls (HC) and youth with major depressive disorder (MDD), frequent MJ use, and both MDD and frequent MJ use. No previously published imaging work appears to have made direct comparisons between reward-processing neurocircuitry in MJ users, MDD, and the combination of MJ use with MDD. We hypothesized that functional brain abnormalities associated with MJ use alone and MDD alone would be exacerbated by their combination. The direct comparison of these groups is intended to help uncover the complex interaction between MJ use and depression and could illuminate combined risk for MJ use in the context of MDD in youth. This information could provide preliminary evidence to help guide clinical recommendations related to marijuana use in youth with MDD. This is an increasingly important topic as more regions in North America consider legalizing marijuana.

## Materials and Methods

### Participants

Approval for the protocol was obtained from the research ethics board at Western Ontario, London, Ontario, Canada. After a complete description of the study to the participants, written informed consent was obtained. Participants were recruited from the local community and through the First Episode Mood and Anxiety Program (FEMAP) in London, Ontario, Canada.

Data were collected from a total of 75 participants. These included 20 HC, 17 youth with MDD, 20 youth with frequent MJ use, and 18 youth with frequent marijuana use and either active or recent major depressive disorder (MDD + MJ). Group assignments were based on the psychiatric diagnosis made by the treating psychiatrist, confirmed by the Structured Clinical Interview for Diagnosis, DSM-IV. Frequent MJ use was assessed by self-report and confirmed by urine screen. Previous research has stratified MJ users in numerous different ways (29, 31). “Frequent” use in this study was defined to be ≥ 4 times per week for at least 3 months preceding the study. The category of “non MJ users” allowed for minimal MJ use since the elimination of any lifetime MJ use was prohibitively restrictive and would decrease the comparability among groups on other variables. Non-significant use was defined as ≤4 times per month for the past year. These limits were chosen to differentiate clinically less serious adolescent/young adult “experimentation,” in the controls, from more consistent MJ use (using the drug more days that not) in the designated MJ users. However, these specifications did not account for the quantity of MJ used on any given day it was used. To confirm group assignments, those participants in the MJ and MJ + MDD groups had positive urine screens for MJ use, and those in the HC and MDD groups had negative urine screens for MJ use.

Prior to fMRI data acquisition, participants were administered the Structured Clinical Interview for DSM Disorders (SCID-I), SCID-II Personality Questionnaire, Family History Screen – Subject Version, Hamilton Depression Rating Scale 21-item (HDRS), Bryden Handedness Questionnaire, Adult ADHD Self-Report Scale (ASRS-v1.1), the Snaith-Hamilton Pleasure Scale (SHPS), Trauma History Questionnaire (THQ), Youth Risk Behavior Surveillance System Questionnaire (YRBS), Emotion Regulation Questionnaire (ERQ), Socioeconomic Status and Demographic Questionnaire, Weschler’s Abbreviated Scale of Intelligence (WASI), Weschler’s Memory Scale (WMS-III), Auditory Processing Composite (APC), and Delis–Kaplan Executive Function System [D-KEFS (DK)]. On the day of the scan, the Spielberger state anxiety inventory (STAI) and the Beck Depression Inventory (BDI)-II were administered to account for mood state on scan day. Because of the high co-use of MJ, alcohol, and tobacco, the use of both alcohol and tobacco was also recorded. The Timeline Follow Back was administered on scan day to evaluate the previous 28 days of MJ and alcohol use. Tobacco use in the last month was recorded in the YRBS.

Participants had no history of head injury or serious medical illness (other than psychiatric diagnosis). The participants included in the MDD group met current criteria for a major depressive episode, while those in the MDD + MJ groups met diagnostic criteria for either current or past MDD, and a total of 13 participants were taking psychoactive medications (primarily SSRIs). There were no significant differences in antidepressant use between the MDD and MDD + MJ groups (*p* = 0.09, n.s.). The doses of medication were stable for 3 weeks prior to fMRI data acquisition. The participants included in the HC group had no history of antidepressant use or personal or family history of psychiatric illness as determined by the SCID. No participants in the MJ group met criteria for a current or past depressive episode. Participants were not excluded based on alcohol use.

### Procedure

All magnetic resonance imaging (MRI) was performed using a 3.0 T MRI scanner (Siemens Verio, Erlangen, Germany) at the Lawson Health Research Institute, using a 32-channel phased array head coil (Siemens). Whole-brain T1-weighted anatomical images with 1mm isotropic spatial resolution were acquired and used as reference for spatial normalization of the data, and to select the orientation of functional MRI images 6° coronal to the AC–PC plane. Music was presented during functional image acquisition (SereneSound audio system and headset). Functional MRI scans consisted of a single-shot echo-planar (T2*-weighted) pulse sequence (volume acquisition time 3 s; 60 slices; 2 mm thick; voxels size 2 mm^3^ isotropic, FOV 25.6 cm × 25.6 cm) for a total time of 6 min per series (two automatically discarded steady-state volumes and 114 brain volumes). Two series were acquired for a total of 228 functional volumes collected for each participant.

#### Music listening paradigm

For each participant, a preferred and neutral piece of instrumental music was determined utilizing ratings on a standardized scale. Prior to scanning, participants were asked to bring in a selection of their own preferred instrumental music, and the preferred music for each participant was selected from these options. This methodology has been utilized in our previous study (27). A bank of neutral music was built from the preferred selections of participants such that a piece of music assessed to be preferred by one participant was a choice of neutral music for the other participants. The earliest participants could choose from a bank of preferred music identified in our prior study using this paradigm in a similar aged participant population (27).

Before scanning, participants listened to both their preferred and neutral music to ensure their subjective experience of them was appropriate for each category. This also familiarized participants to the neutral music to reduce novelty effects. For each piece of music, participants were asked: (1) how much did you like the neutral/preferred music you just heard on a scale from 0 to 100; (2) how much would you like to hear this neutral/preferred music again on a scale from 0 to 100; and (3) to rate the neutral/preferred music you just heard on a scale from −100 to +100.

Each 6-min functional MRI scan consisted of alternating 90-s blocks of music (either preferred or neutral), followed by 60-s blocks of rest (silence). Participants heard two blocks of music (one preferred and one neutral) in each of the two functional scans. The order of the music presentation was randomized across participants. Immediately after fMRI data acquisition, but while still in the MRI machine, subjective enjoyment of the passive music listening was assessed by asking participants to rate each of the music stimuli, using the same standardized scale described above.

### Data analysis

All fMRI data underwent pre-processing including motion correction (INRIAlign), spatial normalization into the standard Montreal Neurological Institute space, and spatial smoothing with a 3D Gaussian kernel with a 8 mm full width at half-maximum radius in preparation for statistical analysis (SPM8, http://www.fil.ion.ucl.ac.uk/spm). In addition, Artifact Detection Tools (ART) software was utilized and those participants with 5 or greater identified artifacts were excluded, carrying forward 61 participants for further analysis. The small number of artifacts remaining was primarily movement related. This was addressed by utilizing six realignment parameters calculated during pre-processing and entered as regressors of no interest during statistical analysis to remove residual motion artifacts. Remaining were 17 HC, 15 MDD, 15 MJ, and 14 MDD + MJ participants. In the MDD + MJ group, 10 met current criteria for current MDD and 4 for past MDD. All statistical analyses were performed using SPM8. Individual participant SPM contrast maps were calculated using the general linear model framework. For each participant, the first 45 s of each music block was modeled using a standard boxcar design convolved with a canonical hemodynamic response function. The first 45 s was used to capture the peak activation related to the task that we hypothesized was most reflective of the reward component of the preferred music. Each music condition block was then compared to the rest condition. These participant-specific maps were then carried forward into a whole brain, second level (between participants; random effects) analysis of covariance (ANCOVA) with two factors involving four groups (HC, MDD, MJ, MDD + MJ) and two music types (preferred, neutral). Sex was included in our model as a regressor of no interest to account for differences in sex composition between groups. Correction for multiple comparisons are as indicated below for each analysis.

Additionally, we performed a whole brain, multiple regression analysis, which incorporated two predictors of interest, MJ use during the past 28 days, and score on the BDI, to examine the relationship between task-related activation and scores on these measures.

## Results

### Questionnaires

Table 1 shows demographic and clinical variables for all participants for whom individual test scores were available (degrees of freedom listed), separated by participant group. There were significant differences between groups in sex composition, with more males than females in both MJ subgroups, but no significant differences existed in age among groups. For age of first MJ use, 13 HC participants and 6 MDD participants had never used MJ, while conversely, 8 MJ participants (4 missing data) and 13 MDD + MJ participants had started using MJ before the age of 17. There were significant differences across groups (Pearson Chi-square = 34.8, df = 9, *p* < 0.0005). With regard to alcohol use, 4 participants had never used alcohol (2 HC and 2 MDD). The mode for age of onset of alcohol use in the HC group was equal between age 15–16 and over 17; the mode for MDD participants was age 17 or over, while for both the MJ (2 missing data) and MDD + MJ participants, it was 13–14 (n.s.).

**Table 1:** **Demographic and clinical variables**.

	Count or mean (SD)				Statistic	*p*-Value
	HC (*n* = 17) a	MDD (*n* = 15) b	MJ (*n* = 15) c	MDD + MJ (*n* = 14) d		
**DEMOGRAPHICS**						
**Gender**						
Male	6	2	10	10	χ^2^[3, 61] = 13.56	0.004
Female	11	13	5	4		
Age at scan (SD)	20.0 (1.1)	19.7 (2.1)	20.2 (1.3)	19.9 (1.7)	*F*[3,57] = 0.18	0.911
Mother education^a^	5.4	5.6	5.5	5.7	χ^2^[15, 52] = 12.78	0.620
Father education^a^	5.4	5.5	5.7	5.1	χ^2^[18, 52] = 13.58	0.756
**CLINICAL VARIABLES, MEAN (SD)**						
**Depression** (*Bonferroni corrected threshold p* < *0.017*)						
BDI	2.6 (3.5)	27.9 (8.9)	7.0 (7.6)	23.1 (14.3)	*F*[3,57] = 26.69	<0.001 (a ↔ b,d; b ↔ c, c ↔ d)
HDRS	0.4 (0.7)	14.0 (3.4)	2.2 (3.9)	13.5 (9.2)	*F*[3,56] = 28.73	<0.001 (a ↔ b,d; b ↔ c, c ↔ d)
SHPS	20.8 (6.4)	31.7 (5.4)	21.7 (3.0)	29.8 (6.6)	*F*[3,57] = 17.04	<0.001 (a ↔ b,d; b ↔ c, c ↔ d)
**Substance use** (*Bonferroni corrected threshold p* < *0.01*)						
Lifetime MJ use^b^	1.8 (1.6)	2.4 (1.5)	6.8 (0.4)	6.9 (0.4)	*F*[3,54] = 60.87	<0.001 (a,b ↔ c; a,b ↔ d)
MJ use past month	0 (0)	0.2 (0.8)	22.0 (6.2)	20.5 (9.2)	*F*[3,57] = 68.95	< 0.001 (a,b ↔ c; a,b ↔ d)
Lifetime alcohol use^b^	5.0 (1.8)	3.8 (2.0)	6.6 (0.8)	6.1 (0.8)	*F*[3,55] = 5.47	0.002 (b ↔ c,d)
Alcohol use past month	14.5 (19.6)	5.1 (9.5)	34.5 (27.0)	25.6 (54.4)	*F*[3,57] = 1.08	0.367
Tobacco use past month (days)^c^	1.1 (0.5)	1.2 (0.4)	2.5 (2.0)	2.9 (2.2)	*F*[3,55] = 4.83	0.005 (a,b ↔ d)
**Other** (*Bonferroni corrected threshold p* < *0.01*)						
Adult ADHD self-report – scale A	1.4 (1.5)	2.9 (1.7)	2.4 (1.5)	2.9 (1.9)	*F*[3,55] = 3.05	0.036
Trauma history questionnaire	1.8 (1.4)	1.7 (1.7)	2.9 (2.9)	4.8 (4.5)	*F*[3,55] = 4.29	0.009 (a,b ↔ d)
Speilberger state anxiety scale	30.2 (8.4)	48.7 (12.0)	35.0 (10.5)	47.9 (11.5)	*F*[3,56] = 11.63	<0.001 (a ↔ b; b ↔ c; a,c ↔ d)
Emotion regulation reappraisal	31.5 (5.1)	25.4 (5.1)	30.1 (7.4)	24.3 (8.1)	*F*[3,57] = 5.10	0.003 ( a ↔ b; c ↔ d)
Emotion regulation suppression	9.5 (3.3)	12.4 (3.4)	8.6 (4.5)	13.1 (3.5)	*F*[3,57] = 6.80	0.001 (a ↔ b; b ↔ c; c ↔ d)
**COGNITION, MEAN (SD)**						
**Weschler abbreviated scale of intelligence (WASI)**	111.2 (10.8)	110.1 (10.3)	105.2 (15.6)	104.6 (6.3)	*F*[3,57] = 1.36	0.264
**Weschler memory scale III** (*Bonferroni corrected threshold p* < *0.007*)						
General memory	55.1 (5.9)	55.5 (8.3)	51.0 (8.5)	49.6 (8.5)	*F*[3,57] = 0.86	0.466
Working memory	22.2 (5.4)	19.8 (4.8)	21.7 (3.8)	21.9 (2.7)	*F*[3,57] = 0.68	0.568
Immediate memory	43.6 (5.8)	41.9 (6.8)	39.4 (7.5)	37.9 (7.0)	*F*[3,57] = 1.33	0.273
Single trial
Learning percentile	52.6 (31.7)	59.0 (29.3)	51.3 (28.9)	46.2 (30.1)	*F*[3,57] = 0.17	0.917
Learning slope
percentile	53.8 (29.8)	44.0 (27.5)	44.8 (20.9)	41.4 (23.7)	*F*[3,57] = 0.64	0.590
Retrieval
percentile	48.8 (24.2)	53.2 (27.7)	40.4 (20.8)	50.0 (30.3)	*F*[3,57] = 0.40	0.753
Retention
percentile	66.3 (21.1)	74.3 (20.3)	65.5 (22.6)	54.6 (33.4)	*F*[3,57] = 1.44	0.239
**Delis-Kaplan executive functioning system, mean (SD)** (*Bonferroni corrected threshold p* < *0.017*)						
Letter fluency						
Total correct	13.3 (3.6)	10.5 (3.1)	13.3 (4.0)	14.2 (3.6)	*F*[3,57] = 1.87	0.144
Category fluency						
Total correct	14.5 (4.1)	12.5 (3.7)	14.1 (3.4)	13.6 (4.0)	*F*[3,57] = 0.74	0.534
Category switching						
Total correct	13.6 (4.5)	13.1 (2.9)	13.6 (2.4)	12.0 (3.4)	*F*[3,57] = 0.60	0.617

*HC, healthy controls; MDD, major depressive disorder; MJ, marijuana; MDD + MJ, depression and marijuana users; BDI, beck depression inventory; HDRS, Hamilton Depression Rating Scale; SHPS, Snaith-Hamilton Pleasure Scale*.

*^a^1 = less than grade 7, 2 = grade 9, 3 = part of high school (grade 10/11), 4 = high school grad, 5 = part college or specialized training, 6 = college/university grad, 7 = graduate professional training (graduate degree)*.

*↔ indicates a statistical difference between variables in columns (a,b,c,d) on either side of symbol*.

*^b^YRBS (1 = never, 2 = 1–2 times, 3 = 3–9, 4 = 10–19, 5 = 20–39, 6 = 40–99, 7 = > 100)*.

*^c^YRBS (1 = never, 2 = 1–2 days, 3 = 3–5, 4 = 6–9, 5 = 10–19, 6 = 20–29, 7 = all 30 days)*.

Interestingly, tobacco use in the last month and MJ use in the last month were correlated only in the non-marijuana users (Spearman’s *r* = 0.46, *p* = 0.008, two-tailed, *N* = 32), perhaps due, in part, to the lack of range in marijuana ratings. Marijuana smokers in both depressed and non-depressed youth showed no correlations among MJ, alcohol, or tobacco use in the last month.

In Table 1, an ANCOVA shows group differences in participant responses to the questionnaires using sex as a covariate of non-interest. Significant group differences were present for variables such as the BDI, HDRS, SHPS, and rates of recent MJ use, as expected. Significant differences among groups on a number of other measures were also present. The MDD + MJ group scored higher on lifetime trauma exposure than either of the two non-marijuana using groups. The two depressed groups scored higher than the two non-depressed groups for anxiety. The non-depressed groups had lower suppression scores on emotion regulation indicating less maladaptive coping than the two depressed groups while the HC had higher reappraisal scores than MDD, and MJ had higher reappraisal scores than MDD + MJ, indicating better adaptive coping. There were no significant differences on the ADHD measure across groups. Likewise, there were no differences on any of the cognitive measures across groups.

### Music ratings

An ANOVA with two factors, group (HC, MDD, MJ, MJ + MDD) and music type (neutral, preferred), found no significant difference in music ratings between groups (*F* = 0.87; *p* < 0.52).

### ANCOVA

**Figure 1:**
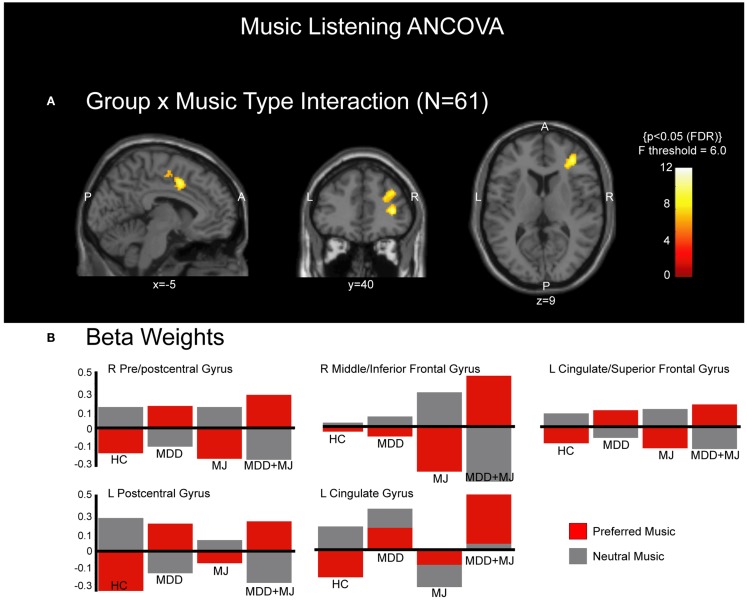
**ANCOVA group × music interaction**. **(A)** shows clusters of activated voxels in the sagittal, coronal, and transverse perspectives overlaid on a normalized T1-weighted anatomical image at MNI planes indicated. P = posterior, A = anterior, L = left, R = right. **(B)** shows the beta weights associated with each identified cluster that met statistical significance, separated by music type and by group.

**Figure 2:**
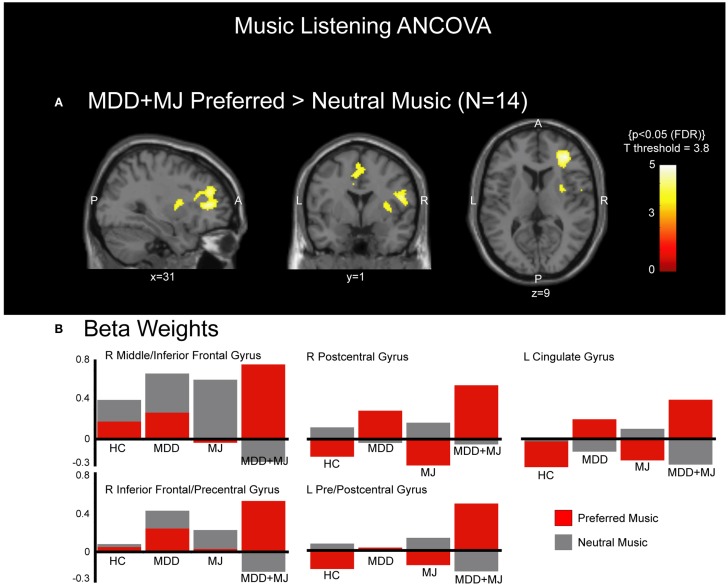
**ANCOVA *post hoc***. **(A)** shows clusters of activated voxels, which had significantly more activation for preferred compared with neutral music in the MDD + MJ group in the sagittal, coronal, and transverse perspectives overlaid on a normalized T1-weighted anatomical image at MNI planes indicated. P = posterior, A = anterior, L = left, R = right. No other *post hoc* comparisons met statistical correction. **(B)** shows the beta weights associated with each identified cluster that met statistical significance, separated by music type and by group.

An ANCOVA (SPM8, http://www.fil.ion.ucl.ac.uk/spm) was performed (*n* = 61) to examine differences among HC, MDD, MJ, and MDD + MJ groups in the passive music listening task. We identified no main effect of group or music type. However, a significant group × music type interaction was present (*F* = 8.42; *p* < 0.05; FDR) as shown in Figure 1A with beta weights illustrated in Figure 1B and regions identified in Table 2. Figure 2 shows the results of *post hoc* comparisons indicating significant differences between preferred and neutral music in the MDD + MJ group, with the preferred music having significantly greater activation (*T* = 3.8; *p* < 0.05; FDR-cluster) in a number of areas including the right middle frontal gyrus, right claustrum (extending into the putamen), and dorsal anterior cingulate (see Table 2). We performed all possible *post hoc* comparisons and no other significant differences were identified within the MDD, MJ, or HC groups or between the groups.

**Table 2:** **Peak activations**.

Region	*k*	Peak co-ordinates (MNI) *x, y, z*	Degrees of freedom and *T* or *F*-scores	*z*-Scores
**ANCOVA INTERACTION GROUP × MUSIC TYPE**				
R pre- and postcentral gyrus (BA 4, 3)	934	56, −14, 40	*F*(3, 113) = 11.95	4.81
L postcentral gyrus (BA 3)	489	−56, −24, 42	*F*(3, 113) = 11.41	4.69
R middle and inferior frontal gyrus (BA 10, 46)	352	38, 40, 8	*F*(3, 113) = 10.73	4.53
L cingulate gyrus (BA 32)	277	−6, 6, 44	*F*(3, 113) = 10.77	4.54
L cingulate and superior frontal gyrus (BA 24, 6)	183	−18, −6, 48	*F*(3, 113) = 9.65	4.26
***POST HOC* MDD + MJ**				
Preferred > neutral
R middle and inferior frontal gyrus (BA 10, 46)	651	38, 40, 8	*T*(1, 113) = 4.90	4.66
R postcentral gyrus (BA 3)	387	54, −18, 36	*T*(1, 113) = 4.72	4.50
L pre- and postcentral gyrus (BA 3, 6)	339	−44, −8, 48	*T*(1, 113) = 4.01	3.87
L cingulate gyrus (BA 24)	375	−6, 4, 46	*T*(1, 113) = 4.62	4.41
R inferior frontal and precentral gyrus (BA 9, 6) extending into claustrum and putamen	455	52, 0, 20	*T*(1, 113) = 4.32	4.14
**MULTIPLE REGRESSION**				
Positive correlation with MJ use
R anterior cingulate (BA 24, 25)	1166	12, 32, 10	*T*(1, 11) = 5.52	3.74
R hypothalamus	–	6, 0, −18	*T*(1, 11) = 5.09	3.58
R cingulate and medial orbital gyrus (BA 32,11)	–	8, 24, −12	*T*(1, 11) = 4.74	3.43
Negative correlation with BDI				
R precentral gyrus (BA 4)	790	52, −16, 24	*T*(1, 11) = 5.74	3.83
R insula (BA 13)	–	38, −8, 16	*T*(1, 11) = 4.96	3.52
R postcentral gyrus (BA 3)	–	48, −22, 32	*T*(1, 11) = 4.53	3.33

*L, left; R, right; BA, Brodmann area, MNI, Montreal Neurological Institute co-ordinate space; *k* = number of voxels in a cluster*.

### Multiple regression

**Figure 3:**
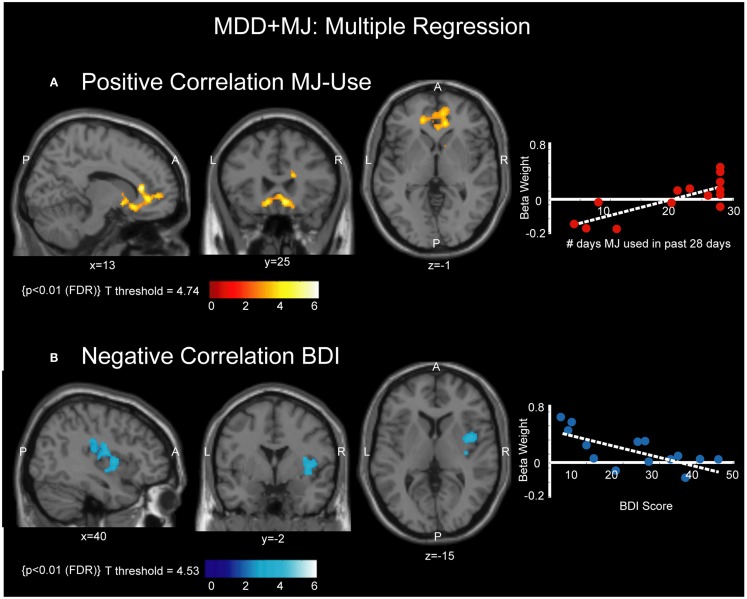
**Correlations between MJ use, BDI score, and activation**. **(A)** Left panel shows voxels with significant positive correlation with MJ use, overlaid on a normalized T1-weighted anatomical image at MNI planes of view indicated to clarify localization. P = posterior, A = anterior, L = left, R = right. **(A)** Right panel shows a scatter plot indicating the relationship between individual participant beta weights of the identified voxels and the number of times they used MJ in the past 28 days. Line of best fit plotted in white. **(B)** Left panel shows voxels with significant negative correlation with BDI, overlaid on a normalized T1-weighted anatomical image at MNI planes of view indicated to clarify localization. P = posterior, A = anterior, L = left, R = right. **(B)** Right panel shows a scatter plot indicating the relationship between individual participant beta weights of the identified voxels and BDI score. Line of best fit plotted in white.

Figure 3 and Table 2 show the results of a multiple regression analysis examining task-related music listening activation (preferred music) in the MDD + MJ group and individual participant scores on MJ use (total MJ used during the past 28 days) and depression rating on the day of the scan (BDI). Figure 3A shows voxels with a significant positive correlation with MJ use (*T* = 4.74; *p* < 0.01; FDR-cluster). These voxels are primarily located in regions localized to the medial frontal cortex, extending from the medial orbital gyrus (BA 11) and the ventral anterior cingulate cortex (ACC) (BA 24) to the dorsal ACC (BA 32), and including the subgenual ACC (BA 25) (described in Table 2). No voxels were identified that showed a significant negative correlation with MJ use. Figure 3A also shows a scatter plot indicating the relationship between individual participant beta weights of the identified voxels and the number of times they used MJ in the past 28 days. Figure 3B shows voxels with a significant negative correlation to scores on the BDI (*T* = 4.53; *p* < 0.01; FDR-cluster). These voxels are located in the insula, extending into the precentral gyrus (BA 4) and the postcentral gyrus (BA 3), described in Table 2. No voxels were identified that showed a significant positive correlation with scores on the BDI. Figure 3B right panel shows a scatter plot indicating the relationship between individual participant beta weights of the identified voxels and scores on the BDI.

## Discussion

The present study used a passive music listening paradigm to examine reward-processing differences in fMRI activation among four groups of youth: HC, frequent MJ users, youth with MDD, and frequent MJ users with MDD. There were several significant group differences including sex, anxiety, emotion regulation style, trauma history, lifetime alcohol use, and past 30 day tobacco use, in addition to the expected differences in depression symptoms and MJ use. Regarding anxiety, the two depression groups had similarly high scores, and likewise, for emotion regulation style, it was the two depression groups that stood out as the most maladaptive compared to the HC and MJ groups. There were no significant group differences in alcohol use in the past month, but the MDD group had less lifetime alcohol use than the MJ or the MDD + MJ groups. In addition, the MDD and MDD + MJ groups included youth taking medication for depression and though medications did not differ between these groups, the HC and MJ alone groups were medication free. All groups reported similarly positive ratings to their preferred music and similarly neutral ratings to the “neutral” music that they heard during fMRI data aquisition.

We identified a significant group by music type interaction but no main effect of group or music type (while covarying for differences in sex across groups). *Post hoc* analysis showed that the interaction was driven by significant differences between preferred and neutral music in the MDD + MJ group in voxels localized to the middle and inferior frontal gyrus, claustrum, putamen, and in the dorsal ACC. These differences between preferred and neutral music were specific to this group, and occurred in the absence of any differences between the groups in subjective music ratings. In addition, we found a significant positive correlation between individual participant scores of MJ used and fMRI activation during the preferred music condition in regions localized to the medial frontal cortex, extending from the medial orbital gyrus (BA 11) and the ventral anterior ACC (BA 24) to the dorsal ACC (BA 32). No voxels were identified that showed a significant negative correlation with MJ use. In contrast, we identified a significant negative correlation between individual participant scores on the BDI and fMRI activation in the insula, extending into the precentral gyrus (BA 4) and the postcentral gyrus (BA2). No voxels were identified that showed a significant positive correlation with scores on the BDI.

The increased BOLD activation identified during the preferred music condition in the MDD + MJ group suggests a relative hyperactivation in PFC and striatal regions during a non-drug rewarding stimulus that did not involve decision making or other cognitive processing. These findings were specific to the MDD + MJ group, and no other group showed this pattern of significantly increased activation. Frequent cannabis users have been shown to demonstrate higher fMRI activation to overt cannabis-related cues in the ventral tegmental area, orbital frontal cortex, ACC, and striatum (32). Subliminal cannabis cues presented using backward masking have also been shown to activate reward neurocircuitry in cannabis-dependent individuals (33). However, debate remains regarding the processing of natural, non-drug rewards. Our results suggest similar activation in PFC areas in response to non-drug rewards in the context of both MDD and MJ use in youth.

Work by Nestor and colleagues (34) have shown that chronic cannabis users have significantly more right ventral striatum BOLD activation during non-drug reward anticipation. The authors suggest chronic cannabis use may modulate reward processing through sensitizing mesolimbic circuits, creating a hypersensitivity to other, non-drug rewards. Our results showing increased BOLD activation in the putamen, ACC, and PFC, all known to be areas involved in reward processing, end support to this hypothesis. However, it was only when MJ use was combined with MDD in our study that this pattern emerged. This may be related to the relatively recent onset of drug use in our participant group, with an average age of first MJ use between 15 and 16 years, and an average age of 20 years at data acquisition. Any mesolimbic hypersensitivity that develops with repeated cannabis exposure may have been more subtle in our participants than in older, chronic cannabis using populations. Another possibility is that previous studies may not have accounted for mood symptoms or depression diagnosis in the investigation of MJ users. It may be that some MJ using participants in prior studies would be categorized as MDD + MJ if mood were evaluated.

Certainly, a large body of evidence exists suggesting altered reward-related neurocircuitry in depressed individuals. Abnormalities have been demonstrated in reward-related brain regions in response to rewarding stimuli in both adults and youth suffering from depression (14, 15, 35). One study showed a normalization of hypo-reactivity of reward-related neurocircuitry in reward-based trials after depressed patients became stably medicated on an SSRI (36).

Our previous work utilizing a passive music listening task in MDD showed decreased BOLD activation in MDD patients to favorite versus neutral music in the nucleus accumbens/ventral striatum and medial orbital PFC (27). The current results did not demonstrate this pattern in MDD alone versus HC youth. One possible reason is that the previous work did not account for rate of marijuana use. As a result, frequent MJ users who did not meet criteria for abuse or dependence, which in our experience is common in youth with mood complaints (37), were not excluded from the previous study. The results of the current study suggest a relationship between MJ use and MDD that underscores the importance of accounting for both depression and rate of cannabis use in studies of functional brain differences in youth.

Of importance, the beta weigh charts illustrate a potentially interesting pattern. The configuration of the BOLD response to preferred music in the MDD + MJ group is more similar to the MDD group than to either the HC or MJ groups in all brain regions where differences were found, and these differences between preferred and neutral music were larger than in the MDD participants and often in the opposite direction to the HC and MJ groups’ responses. This suggests that there are unique characteristics in the combination of MJ and MDD. Two possibilities could be implied by these results. First, it may be that the use of MJ in the context of MDD exaggerates abnormal brain modulation found in MDD alone, perhaps by increased sensitization of mesolimbic circuits, creating an increased activation to the preferred music condition. Or, alternatively, the use of MJ in the context of previous/current MDD may be an attempt to self-medicate the anhedonia associated with MDD, with a paradoxical “overshoot” of response to the rewarding stimulus. If MJ has such effects on mesolimbic circuits, it might appeal to individuals who have intrinsic difficulties with reward processing or negative biases, as is present in MDD. Regardless of the causal associations, youth with MDD may be particularly susceptible to the effects of MJ on the brain based on these findings. It is impossible to tell from the current study design if this increased perturbation of the mesolimbic system in the MDD + MJ group would also hold true for youth at risk for MDD but without current or previous symptoms, posing an interesting question for future research. Further work is required to determine the mechanism accounting for this interaction between MJ use and MDD.

The significant positive correlation we identified between activation in the medial frontal cortex and number of times MJ was used in the past 28 days in the MJ + MDD group (Figure 3) suggests that brain activation in this region is scaling positively with this variable, though there could be some “ceiling effect” related to the number of youth who were smoking 30 or more times (see scatterplot, Figure 3). These areas comprised multiple regions of cortex extending from the medial orbital gyrus (BA 11) and ventral anterior ACC (BA 24) to the dorsal ACC (BA 32), and included the subgenual ACC (BA25) and the hypothalamus. Previous work investigating non-intoxicated MJ users has described increased rCBF in the ACC in cannabis users (38), and a correlation between the total number of smoking episodes per week as well as overall cannabinoid level and activation in the cingulate cortex during the viewing of masked emotional face stimuli (39). In addition, recent work by Harding and colleagues has shown that increased connectivity between the ACC and a number of areas including the PFC and anterior insula correlates positively with lifetime exposure to cannabis (40). A number of other studies of cannabis use have shown task-related fMRI findings of modulation in the ACC, some showing increases relative to HC (39), and others showing decreases (41–43). We did not identify activation in the striatum correlating positively with MJ use, specifically, which would have been predicted from the incentive salience hypothesis (44), a theory suggesting that repeated exposure to drugs of abuse may lead to hyperactivity in that region. Again, however, the participants studied here were young and in the early stages of cannabis use, as well as being depression sufferers. There are direct projections from the subgenual PFC, including BA 25 as found here, to the ventral striatum, including the nucleus accumbens (45). It may be that the paradigm we used preferentially activated the PFC rather than ventral striatal regions, or it could be that the alterations in ventral striatum develop only after extended years of MJ use. The positive correlation between BOLD signal and MJ use in the medial PFC, including the subgenual ACC (BA25) is of particular note given the importance of this region from brain imaging studies of MDD (46–48), most of which find hyperactivation in this region. This lends some support to the possibility that the use of MJ in the MDD + MJ subgroup was associated with greater abnormality in this critical area of limbic regulation known to be abnormal in MDD, and reinforces the finding that combined MJ use with MDD was associated with increased alteration in brain function in some regions.

In addition, we identified a significant negative correlation between scores on the BDI and activation in the insula, extending through the claustrum into the putamen, and in the precentral (BA 4) and postcentral gyri (BA2), as shown in Figure 3B. This indicated that higher depression scores were associated with less activation in these brain regions. The critical role of the insula in depressive symptoms is supported by a body of evidence including previous work that utilized PET imaging and principle components analysis of the BDI and found the psychomotor-anhedonia symptom cluster correlated with lower absolute metabolism in several areas including the insula, claustrum, and caudate/putamen (49). A large meta-analysis has identified the insula as part of a network of brain regions that are hypoactive in depressed participants but increase their activation with treatment (50).

It should be noted that there was a strong correlation between state anxiety and the BDI in the entire group (Spearman’s *r* = 0.77, *p* < 0.0005) and in the MDD + MJ group alone (Spearman’s *r* = 0.79, *p* = 0.001). So, the negative correlation found here could be due to anxiety, which is often comorbid with depressed mood.

Overall, our findings have shown relationships between MJ + MDD and abnormalities in brain regions associated with emotional processing in response to a natural, non-drug reward that was not present in either MDD or MJ groups alone, but which may represent an exacerbation of the negative effects on brain function of MDD alone and/or an ineffective attempt to self-medicate abnormal brain function. Future research may help to clarify this complex relationship. These findings have implications for clinical treatment in that they suggest that the use of MJ in the context of MDD in young adults imposed additional brain abnormalities than either alone. In an era when marijuana products have been suggested by the lay public as a treatment for depression, these results indicate that controlled clinical trials are warranted before such an approached should be promoted.

## Author Contributions

Kristen A. Ford, Richard W. Neufeld, Derek Mitchell, Jean Théberge, Peter Williamson, and Elizabeth A. Osuch contributed to the design of the work; Kristen A. Ford, Jean Théberge, and Elizabeth A. Osuch contributed to the data acquisition. All authors contributed to the analysis and/or interpretation of data for the work and all authors contributed to drafting the work and/or revising it critically for important intellectual content. All authors gave final approval of the version to be published and are in agreement to be accountable for all aspects of the work. Elizabeth A. Osuch oversaw the study and was the Principal Investigator of the project.

## Conflict of Interest Statement

The authors declare that the research was conducted in the absence of any commercial or financial relationships that could be construed as a potential conflict of interest.
